# Genetic interaction between rice *PLASTOCHRON* genes and the gibberellin pathway in leaf development

**DOI:** 10.1186/s12284-014-0025-2

**Published:** 2014-09-18

**Authors:** Manaki Mimura, Jun-Ichi Itoh

**Affiliations:** 1 Graduate School of Agricultural and Life Sciences, University of Tokyo, Tokyo 113-8657, Japan

**Keywords:** Rice, PLASTOCHRON 1, PLASTOCHRON 2, Gibberellin, SLENDER RICE 1

## Abstract

**Background:**

The rice *PLASTOCHRON* (*PLA*) genes *PLA1* and *PLA2* regulate leaf maturation and the temporal pattern of leaf initiation. Although the function of *PLA* genes in the leaf initiation process has been analyzed, little is known about how they affect leaf growth. Previously, we suggested that *PLA1* and *PLA2* function downstream of the gibberellin (GA) signal transduction pathway. In the present study, we examined the phenotype of a double mutant of *pla* and *slender rice 1* (*slr1*), which is a constitutive GA response mutant. By analyzing these double mutants, we discuss the relationship between *PLA*-related and GA-dependent pathways and the possible function of *PLA* genes in leaf growth.

**Findings:**

Single *slr1* and *pla* mutants exhibited elongated and dwarf phenotypes in the vegetative stage, respectively. The stature and leaf size of the *pla1/slr1* and *pla2/slr1* double mutants were intermediate between those of the *pla* and *slr1* single mutants. However, the effects of *slr1* on leaf elongation were markedly suppressed in the *pla1* and *pla2* mutant backgrounds. On the other hand, the change in cell length in the double mutants was almost the same as that in the single mutants. An expression analysis of genes involved in GA biosynthesis and catabolism indicated that feedback regulation functioned normally in the *pla/slr1* double mutants.

**Conclusions:**

Our genetic results confirm that *PLA* genes regulate leaf growth downstream of the GA pathway. Our findings also suggest that *PLA1* and *PLA2* are partly required for GA-dependent leaf elongation, mainly by affecting cellular proliferation.

## Findings

Rice *plastochron* (*pla*) mutants show a short plastochron and small precocious leaves. *PLA1* and *PLA2* encode a cytochrome P450, CYP78A11, and an RNA-binding protein, respectively. They are expressed in leaf primordia and regulate the leaf initiation rate and leaf maturation (Miyoshi *et al.*[2004]; Kawakatsu *et al.*[2006]). Thus, *PLA1* and *PLA2* play important roles in leaf development. Previously, we showed that *PLA1* and *PLA2* function downstream of the gibberellin (GA) signal transduction pathway (Mimura *et al.*[2012]), and that *pla1* and *pla2* plants exhibited reduced sensitivity to GA treatment. In addition, GA treatment induced *PLA1* and *PLA2* expression. In accordance with these results, the expression levels of *PLA* genes were increased in *slender rice 1* (*slr1*), which is a constitutively active GA signaling mutant, and decreased in *slr1-D*, which shows reduced sensitivity to GA. However, genetic evidence for the interaction between *PLA* genes and GA signaling genes is lacking. In the present study, we constructed *pla1/slr1* and *pla2/slr1* double mutants to investigate the genetic relationships between *PLA* genes and the GA signaling pathway.

### Phenotypes of *pla1* and *slr1* double mutants

*slr1* is a constitutive GA response mutant that is caused by a loss-of-function of DELLA, which is a key factor in the repression of GA responses (Ikeda *et al.*[2001]). *slr1* mutants showed elongated leaves and internodes. In contrast, *pla* mutants showed dwarfism and small leaves. *PLA1* encodes the cytochrome P450 family protein CYP78A11, which is a member of the CYP78A subfamily (Miyoshi *et al.*[2004]). Many reports have shown that CYP78A family genes regulate organ growth (e.g., seed or fruit size) in several plant species (Anastasiou *et al.*[2007]; Fang *et al.*[2012]; Chakrabarti *et al.*[2013]; Sotelo-Silveira *et al.*[2013]). It has also been suggested that CYP78A family members are involved in producing an as yet unidentified substance that functions as a mobile growth regulator (Anastasiou *et al.*[2007]; Adamski *et al.*[2009]; Eriksson *et al.*[2010]).

**Figure 1:**
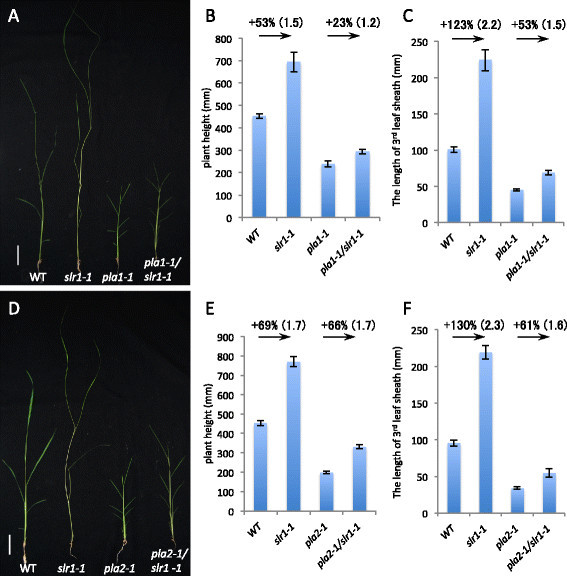
**Phenotypes of the** ***pla1/slr1*** **and** ***pla2/slr1*** **double mutants. (A–C)** Phenotypes of wild-type, *slr1-1*, *pla1-1*, and *pla1-1/slr1-1* plants. **(D–F)** Phenotypes of wild-type, *slr1-1*, *pla2-1*, and *pla2-1/slr1-1* plants. **(A, D)** Seedlings at 3 weeks after germination (DAG). **(B, E)** Plant height at 3 weeks after germination. **(C, F)** Length of the third leaf sheath. The values represent means ± SE (*n* > 5)*.* Fold-increase are shown in parenthesis after% increase. The scale bars indicate 5 cm.

To determine the genetic interaction between *SLR1* and *PLA1*, we generated *pla1/slr1* double mutants by crossing *SLR1* heterozygous plants with *PLA1* heterozygous plants. At the 3-week-old seedling stage, the *pla1/slr1* double mutants showed intermediate phenotypes (Figure 1A–C, Table 1). However, the effects of the *slr1* mutation on plant height and leaf size in the *pla1* background were weaker than those in wild type. The *slr1* plants were 53% taller than the wild-type plants, whereas the height of the *pla1/slr1* double mutant was 23% that of the *pla1* single mutant (Figure 1B). In terms of leaf length, the effect of the *slr1* mutation was much more obvious in the wild-type background than in the *pla1* mutant background. The third leaf sheath of *slr1* was 123% longer than that of wild type, whereas that of the *pla1/slr1* double mutant was only 53% that of the *pla1* single mutant (Figure 1C). These results suggest that *PLA1* activity is partly necessary for leaf elongation in *slr1* mutant plants.

**Table 1:** **Seedling phenotypes of the** ***pla1-1/slr1-1*** **and** ***pla2-1/slr1-1*** **double mutants at 3 weeks after germination**

**Genotype**	**Plant height (mm)**	**Leaf number**	**1st Leaf (mm)**	**2nd LB (mm)**	**2nd LS (mm)**	**3rd LB (mm)**	**3rd LS (mm)**	**4th LB (mm)**	**4th LS (mm)**	**n**
WT	453 ± 11.3	5.6 ± 0.16	19.3 ± 0.6	21.0 ± 1.4	45.7 ± 1.7	79.0 ± 3.9	100.7 ± 3.5	153.7 ± 5.5	166.6 ± 5.4	10
*slr1-1*	693 ± 43.3	4.8 ± 0.17	27.8 ± 2.5	25 ± 2.6	109.5 ± 8.5	186.0 ± 17.5	224.3 ± 14.4	381.2 ± 29.8	321.8 ± 23.1	6
*pla1-1*	238 ± 13.0	7.8 ± 0.16	14.9 ± 0.7	12.6 ± 1.0	27.6 ± 1.5	29.1 ± 1.9	45.0 ± 1.4	35.1 ± 0.8	62.6 ± 1.9	8
*pla1-1/slr1-1*	294 ± 10.8	7.8 ± 0.31	15.0 ± 1.2	10.9 ± 1.5	36.3 ± 3.7	26.9 ± 4.1	69.0 ± 3.2	47.8 ± 3.3	92.3 ± 4.4	8
WT	454 ± 12.3	5.9 ± 0.17	20.4 ± 0.8	20.1 ± 1.0	44.1 ± 1.8	77.3 ± 4.2	95.5 ± 4.1	157.3 ± 4.8	142.5 ± 6.7	11
*slr1-1*	771 ± 25.0	5.0 ± 0.21	30.5 ± 1.7	25.2 ± 1.5	109.7 ± 6.3	162.5 ± 11.9	219.2 ± 9.4	396.7 ± 14.2	344.2 ± 18.2	10
*pla2-1*	199 ± 6.7	9.3 ± 0.15	14.5 ± 0.9	4.6 ± 0.5	23.0 ± 1.6	12.6 ± 1.0	34.0 ± 1.7	27.6 ± 2.2	46.4 ± 1.5	10
*pla2-1/slr1-1*	332 ± 8.9	8.4 ± 0.51	16.8 ± 1.6	4.8 ± 0.9	34.4 ± 4.2	16.8 ± 1.3	54.8 ± 5.7	40.0 ± 4.8	75.4 ± 5.5	5

LB: leaf blade, LS: leaf sheath. The values indicate the means ± SE. n indicate the number of seedlings examined in this study.

**Figure 2:**
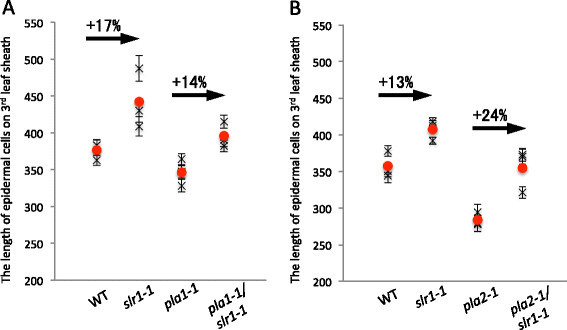
**Length of epidermal cells on the adaxial side of the third leaf sheath. (A)** Epidermal cell length in wild-type, *slr1-1*, *pla1-1*, and *pla1-1/slr1-1* plants. **(B)** Epidermal cell length in wild-type, *slr1-1*, *pla2-1*, and *pla2-1/slr1-1* plants. Crosses indicate average values of the cells in one sample (average ± SE; *n* > 100). Closed circles indicate average values of three independent samples.

Cell size is one of the factors determining leaf size. To clarify how cell size contributes to leaf elongation in *slr1* and *pla1/slr1* double mutants, we compared the lengths of epidermal cells on the adaxial side of the third leaf sheath in each mutant (Figure 2A). Our results indicate that the effects of the *slr1* mutation on cell size were comparable between the wild-type and *pla1* backgrounds. Cell length was increased by 17% in *slr1* single mutant plants and by 14% in *pla1/slr* double mutant plants compared to the corresponding genotypes. These results indicate that *PLA1* contributes mainly to cell proliferation in GA-dependent leaf elongation.

### Phenotypes of *pla2* and *slr1* double mutants

*PLA2* encodes an RNA-binding protein; however, its target RNAs have yet to be elucidated (Kawakatsu *et al.*[2006]). Similar to the *pla1/slr1* double mutants, we examined the phenotype of *pla2/slr1* double mutants. The stature and leaf size of the double mutants were intermediate between those of the *pla2* and *slr1* mutants (Figure 1D–F, Table 1). With regard to plant height, 3-week-old *slr1* and *pla2/slr1* seedlings were 69% and 66% taller than wild-type and *pla2* seedlings, respectively (Figure 1E). With regard to the length of the third leaf sheath, those of the *slr1* and *pla2/slr1* plants were 130% and 61% longer than in the corresponding genotypes, respectively (Figure 1F). These results suggest that *PLA2* is also at least partially involved in GA-dependent leaf elongation.

Next, we measured the length of epidermal cells on the adaxial side of the third leaf sheath. The cells of the *pla2/slr1* double mutant were elongated by 24% compared to the *pla2* single mutant; whereas those of *slr1* were 13% longer than in wild type (Figure 2B). These results indicate that normal GA-dependent cell elongation occurred in the *pla2/slr1* double mutants. Accordingly, the suppression of the *slr1* phenotype in the *pla2/slr1* double mutant may have been due to a reduction in cell number.

### Expression of genes involved in GA biosynthesis and catabolism in *pla/slr1* double mutants

**Figure 3:**
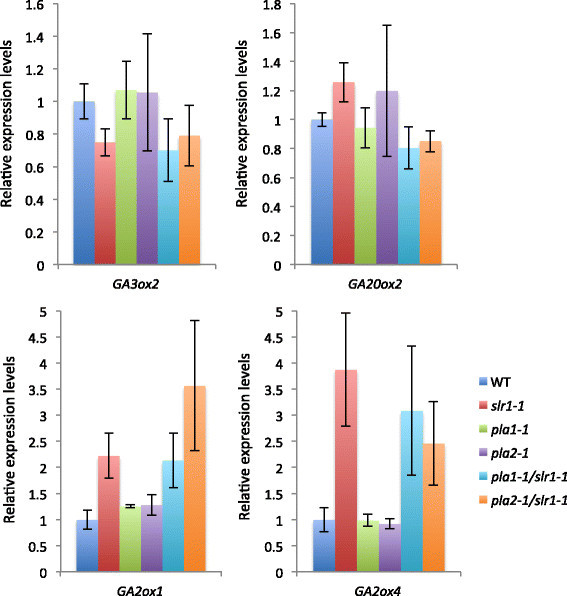
**Relative expression levels of GA biosynthetic (*GA3ox2, GA20ox2*) and GA catabolism (*GA2ox1*,** ***GA2ox4*) genes in wild-type,** ***slr1-1*,** ***pla1-1*,** ***pla2-1*,** ***pla1-1/slr1-1*, and** ***pla2-1/slr1-1*** **plants.** The expression levels in the mutants are represented relative to that in wild type (assigned a value of 1). The values indicate the means of three biological samples ± SE. *Actin1* was used as an internal control. *GA3ox2, GA20ox2*, *GA2ox4* and *Actin1* were quantified using TaqMan probes. *GA2ox1* was quantified by SYBR green. The primers and probes for each gene are listed in Additional file 1: Table S1.

The content of bioactive GA is maintained through feedback regulation (Dai *et al.*[2007]; Olszewski *et al*. [2002]; Yamaguchi [2008]). To investigate whether feedback regulation for GA homeostasis occurred normally in our *pla/slr1* double mutant plants, we examined the expression levels of two GA biosynthetic genes *GA3 oxidase2* (*GA3ox2*) and *GA20 oxidase2* (*GA20ox2*), and two GA catabolism genes *GA2 oxidase1* (*GA2ox1*) and *GA2ox4*, in these mutants by real-time PCR (Figure 3, Additional file 1: Table S1). In *pla1* and *pla2* mutant plants, the expression levels of these GA biosynthetic and GA catabolism genes were comparable to those in wild-type controls. Thus, *PLA1* and *PLA2* do not affect the expression of genes involved in GA metabolism. The expression of *GA3ox2* was slightly decreased in the *slr1* mutant plants, as reported previously (Dai *et al.*[2007]). The expression of *GA20ox2* was not decreased in *slr1* mutant, indicating that the expression of *GA20ox2* may not be under GA feedback regulation in *slr1* mutant. In contrast to *GA3ox2* gene, the expression levels of both the GA catabolism genes in *slr1* mutant were increased compared to wild type. Similar to the levels seen in the *slr1* mutant, *GA3ox2* expression was downregulated and *GA2ox1* and *GA2ox4* expression was upregulated in the *pla1/slr1* and *pla2/slr1* double mutant plants. These results indicate that the feedback mechanism and GA response were normal in the *pla/slr1* double mutants, at least at the transcriptional level.

GA is involved in various developmental processes, including seed germination, stem elongation, flowering, and pollen maturation (Olszewski *et al*. [2002]; Yamaguchi [2008]). Microarray studies have identified several genes involved in the GA pathway (Yazaki *et al.*[2003]; Yang *et al.*[2004]; Jan and Komatsu [2006]). However, the genetic regulation downstream of the GA pathway in leaf development is poorly understood. Previous studies suggested that *PLA* gene products function downstream of GA. In this study, we demonstrated genetic interactions between *PLA* genes and *SLR1*, a central regulator of GA signaling, supporting our previous results.

Our analysis suggests that the intermediate phenotypes of the *pla/slr1* double mutants were probably due to a reduction in cell number. There are two explanations for why the absence of *PLA* functions partly suppressed the *slr1* phenotype. First, *PLA* genes are involved in cellular proliferation in the GA-dependent pathway. Recent studies have indicated that GA promotes not only cell expansion but also cellular proliferation through the regulation of cell cycle inhibitor genes (Achard *et al.*[2009]). In addition, GA controls the transition from cell proliferation to expansion in maize leaves (Nelissen *et al.*[2012]). Thus, GA can influence cell number during leaf development, and it is possible that *PLA* functions are required for cell proliferation rather than cell elongation downstream of the GA pathway. Second, defects in *PLA* genes affect the duration and/or timing of cellular proliferation, resulting in a decrease in the total cell number in the leaves of *pla/slr1* mutants. Previous studies suggested that *PLA1* and *PLA2* genes regulate the rate of leaf maturation (Kawakatsu *et al.*[2006]) and that the small leaves in *pla* mutants were due to precocious leaf maturation. Thus, it is possible that the duration of cell proliferation in developing leaves is insufficient in *pla/slr1* double mutants, resulting in suppression of the *slr1* phenotype.

Our results indicate that *PLA* genes partly regulate leaf size by affecting cell proliferation via the GA-dependent pathway. However, it remains unclear how *PLA* genes regulate cellular proliferation in the GA signaling pathway. Further study is required to clarify the molecular mechanisms underlying the regulatory roles of *PLA* gene expression in GA-dependent leaf development.

## Abbreviations

GA: Gibberellin

PLA: PLASTOCHRON

SLR1: SLENDER RICE1

## Competing interests

The authors declare that they have no competing interests.

## Authors’ contributions

MM performed the experiments and drafted the manuscripts. JI conceived the study and drafted manuscripts. Both authors read and approved the final manuscript.

## Additional file

## Supplementary Material

Additional file 1: Table S1. List of primers used in real-time PCR assays. Click here for file
